# Case Report: Progressive Cholestasis: Severe Phenotype of MEGDEL Syndrome With SATB2-Associated Syndrome

**DOI:** 10.3389/fped.2021.713458

**Published:** 2021-10-01

**Authors:** Yajie Su, Hui Zhang, Huijun Wang, Bingbing Wu, Jiao Yang, Wenhao Zhou, Long Li

**Affiliations:** ^1^Department of Neonatology, Children's Hospital of Fudan University, Shanghai, China; ^2^Department of Neonatology, Children's Hospital of Xinjiang Uygur Autonomous Region, Urumqi, China; ^3^Shanghai Key Laboratory of Birth Defects, Pediatrics Research Institute, Shanghai, China; ^4^Center for Molecular Medicine, Children's Hospital of Fudan University, Shanghai, China

**Keywords:** MEGDEL syndrome, SATB2-associated syndrome, phenotype, next-generation sequencing, follow-up

## Abstract

MEGDEL syndrome and *SATB2*-associated syndrome (SAS) are both rare congenital disorders with poor prognoses caused by gene mutations. We present the case of a 2-day-old girl with an unexplained abnormal liver function, feeding problem, and dystonia. Using next-generation sequencing, we identified two novel mutations in *SERAC1* and a mutation in *SATB2*. Now, she is 15 months old and has the characteristics of SAS, such as downslanting palpebral fissures and delayed primary dentition. Besides the typical phenotypes of MEGDEL syndrome, such as hypertonia, failure to thrive, deafness, and motor regression, she has progressive cholestasis and is prone to high serum lactate after rehabilitation training and hypoglycemia with low ketone under starving conditions. These phenotypes substantially differ from the transient liver function abnormalities and hypoglycemia reported in the literature.

## Introduction

MEGDEL syndrome (3-methylglutaconic acidemia, deafness, encephalopathy, and Leigh-like syndrome) is a rare specific mitochondrial disorder due to mutations in the *SERAC1* gene (NM_032861), which is an autosomal recessive inherited disorder (1). The *SERAC1* gene encodes for a phosphatidylglycerol remodeler that is essential for mitochondrial functions and intracellular cholesterol trafficking (2). The *SATB2*-associated syndrome (SAS) is a recently described syndrome caused by mutaion of *SATB2* gene (NM_015265). It's an autosomal dominantly inherited disorder which characterized by developmental delay with absent or limited speech, behavioral problems, dysmorphic features, and craniofacial abnormalities, including palatal and dental abnormalities (3).

We describe a rare disease progression and a severe phenotype of progressive cholestasis of a girl from birth to 15 months, who was compound heterozygous for *SERAC1* of MEGDEL syndrome and had a mutation in *SATB2* of *SATB2*-associated syndrome.

## Case Description

A female infant was born at 38+ 5/7 weeks gestational age to a 38-year-old mother who had gestational anemia, which was supplemented with oral iron, and gestational diabetes, which was controlled with diet. The mother has a healthy 6-year-old girl but also had a spontaneous abortion and an artificial abortion due to embryo death. The mother's brother had a history of epilepsy and had passed away.

The infant's birth weight, head circumference, and length were 2,830 g (10–25th percentile), 33 cm (25–50th percentile), and 48 cm (10–25th percentile), respectively, with a normal Apgar score. However, she had mild blepharoptosis and there was a 3 cm cleft palate from the palate to the uvula. On day of life (DOL) 2, the infant vomited milk twice accompanied by weakness in sucking. A few hours later, she went into convulsions that involved rowing movements of the limbs. Her blood glucose was 0.7 mmol/L and blood gas analysis revealed anion gap metabolic acidosis (24.69 mmol/L). After providing glucose (8 mg/kg/min) and phenobarbital (10 mg/kg) injection treatment, the convulsions did not occur again, but she became somnolent, developed an unexplained abnormal liver function, and an aggravating increase in serum lactate (16.4 mmol/L). The infant was immediately transferred to our tertiary neonatal intensive care unit (NICU) center on DOL 3 for further newborn care.

**Figure 1:**
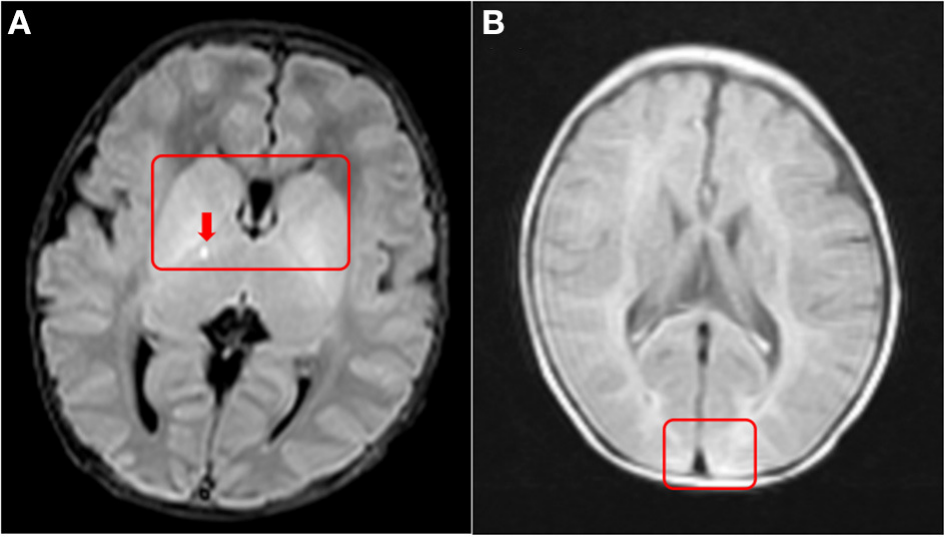
Brain MRI at 10 days and 3 months of age. **(A)** T2W: Red boxes indicate a high signal of basal ganglia, swelling of bilateral lenticular nucleus; Red arrows indicate an abnormal signal point of posterior branch of right internal capsule. **(B)** T2W: Red boxes indicate white matter of occipital lobe has high signal, and the boundary of gray and white matter is not clear.

The infant's serum lactate was normal on DOL 4 after giving intravenous fluids and nasogastric feeding. Blood glucose was normal on DOL 6 without intravenous fluids. The problems during hospitalization were poor neonatal sucking, reduced movements, and unexplained elevated liver enzymes (gamma-glutamyltransferase (GGT) 1,373 mmol/L, total bile acids (TBA) 58.2 μmol/L, aspartate aminotransferase (AST) 132 U/L,prothrombin time (PT) 46.1s, albumin 26.57 g/L) with normal stool color and abdominal ultrasound. In addition, magnetic resonance imaging (MRI) showed high signals on T2 and FLAIR, where the lenticular nucleus was swollen on both sides. There were multiple hyperintensity patches on the dorsal side of the brainstem and posterior limb of the internal capsule near the anterior and posterior horns of the right lateral ventricle (Figure 1). Evoked otoacoustic emission test was not passed on both ears.

**Figure 2:**
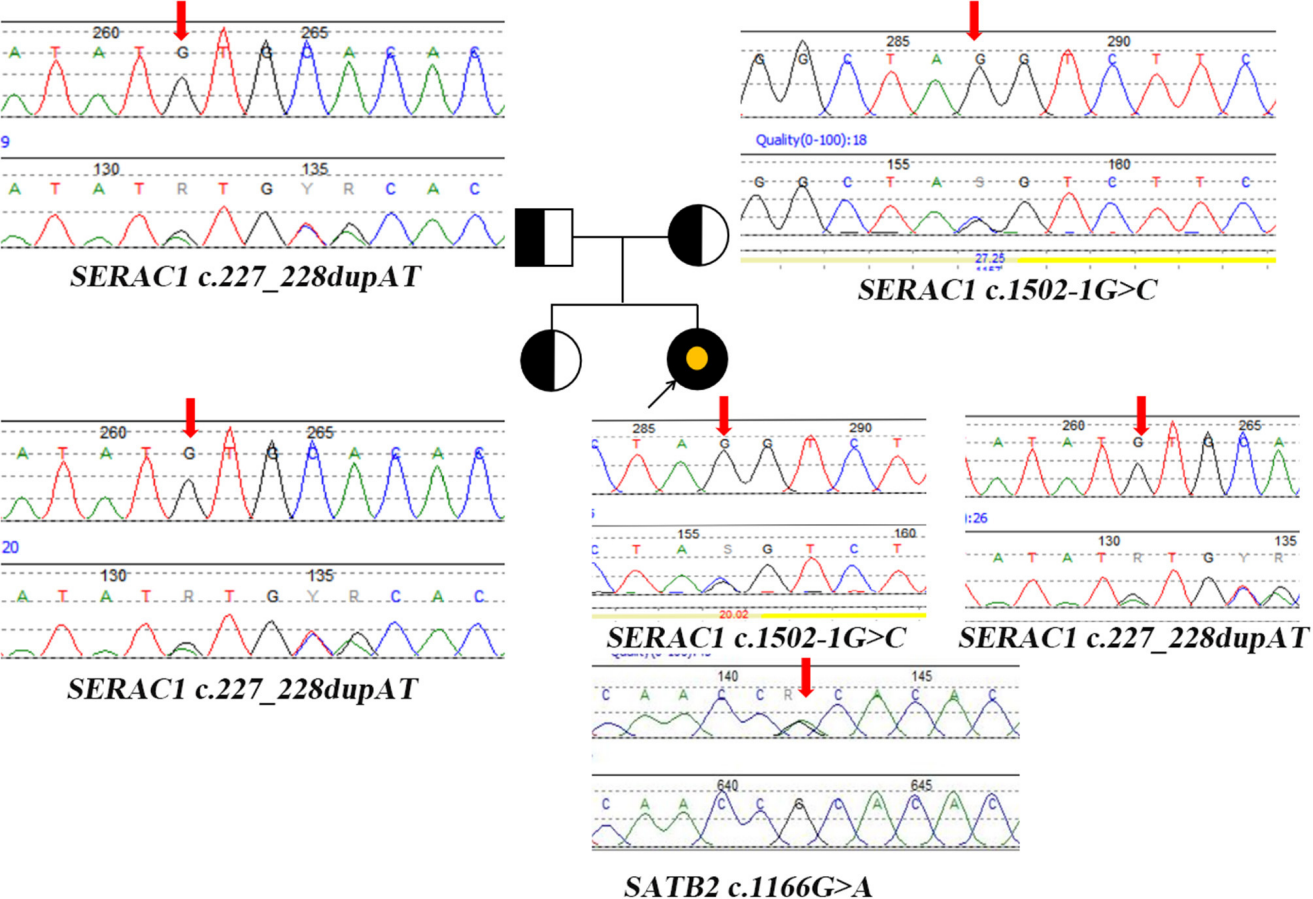
Pedigree diagram and genetic characterization of the family with *SERAC1* and *SATB2*. Parents and sisters are heterozygous carriers of the *SERAC1* gene; The yellow point represents the mutation of *SATB2* gene. Heterozygous mutation (c.227-228dupAT) in *SERAC1* was found in the patient, her father and her healthy sister. Heterozygous mutation (c.1502-1G>C) in *SERAC1* was found in the patient and her mother. Heterozygous mutation (c.1166G>A) in *SATB2* was only found in the patient; Red arrow indicate location of mutation.

Blood tandem mass spectrometry (MS/MS) showed an increase in tyrosine 227.85 μM (25–225 μM). Urine gas chromatography-mass spectrometry (GC/MS) showed an increase in oxalate 57.78 (0–33), 3-methylglutaconic aciduria 4.35 (0–1), and 3-(4-hydroxyphenyl) lactic acid 1122.49 (0–20). Next-generation sequencing and Sanger sequencing detected two compound heterozygotes of *SERAC1* (c.1502-1G>C(likely pathogenic) from the maternal side, c.227-228dupAT(pathogenic) from the paternal side) and a *de novo* variant of *SATB2* (c.1166G>A) (likely pathogenic) (Figure 2). The quality control of WES data analysis and procedure of variant annotation refers to pipeline version 2 of Children's Hospital of Fudan University (4). We classified the pathogenicity of the identified variants according to the ACMG guideline, the details showed in (Supplementary Table 1).

On DOL 17, the infant was discharged from hospital with a nasogastric tube. After symptomatic treatment, the high GGT at admission had dropped to 264 mmol/L and the PT returned to normal (16.2 s). She experienced slow weight gain and increasing TBA, which was unmanageable by drugs after discharge. When she was 3 months old, there was a continuous mild increase in methionine (Met) for 2 months. After providing Met-free formula milk, the level of Met reduced to normal, and her motor development improved. When she was 12 months old, she had a high fever of 39°C. Afterward, her gross and fine motor regressed to the level of 5 months. Now, she is 15 months old with four primary teeth and has completed a cleft palate repair surgery in order to improve her nutritional status. When she was discharged from the hospital in the first time, the GGT were maintained between 430 and 470 mmol/L, the AST were maintained between 62 and 87 U/L and albumin were maintained between 31.4 and 36.9 g/L. In addition, PT (15.1 s) and international normalized ratio (1.31) was slightly elevated at 12 months of age. Only bile acids were continuously increasing. Because she had normal stool color, normal skin color, and normal abdominal ultrasound, we excluded the extrahepatic biliary atresia and choledochal cyst and did not perform liver biopsy. She is susceptible to higher serum lactate after rehabilitation training and hypoglycemia with low ketone under starving conditions (Table 1).

**Table 1:** Follow-up of the infant from birth to 15 months of age.

**Age**	**0 d**	**3 d**	**17 d**	**3 m**	**6 m**	**9 m**	**12 m**	**15 m**
Event	Born	NICU	Discharge	Rehabilitation training	Intravenous injection of levocarnitine	Free-Met milk powder	High fever of 39°C+first primary teeth	Cleft palate repair surgery
Weight (g)	2,830 (<25th)	2,630 (<25th)	2,780 (<25th)	4.5 (<5th)	5,500 (<2th)	6,200 (<2th)	7,000 (<2th)	7,800 (<5th)
Height (cm)	48 (<25th)	48 (<25th)	48 (<25th)	53 (<2th)	61 (<2th)	67 (<10th)	72 (<25th)	77 (<50th)
Feeding	Oral feeding	Intravenous fluids and nasogastric feeding	Oral+nasogastric feeding	Oral feeding formula milk	Oral feeding formula milk	Supplementary food+free-Met formula milk (2 months)	Supplementary food+formula milk	Supplementary food+ formula milk
Bile acid (μmol/L)	\	58.20	36.31	149.31	492.20	526.67	386.54	325.7
Lactate (mmol/L)	\	9.25	2.03	2.10	1.90	2.70	5.82	3.60
Others	\	Metabolic acidosis, elevated liver enzymes	Elevated liver enzymes	Elevated liver enzymes	Elevated liver enzymes, cholesterol (3.3 mmol/L)	Hypoglycemia with low ketone	Cholesterol (2.49 mmol/L)	\
	\	aEEG:Intermittent low amplitude in both hemispheres (10–15 μV)	Abdominal CT: the density of liver parenchyma was reduced. The bilateral renal pelvis was slightly dilated	MRI:T2 showed a lower signal of white matter	\	Indexes related to islet function were normal	\	\
Central Nervous System	Normal	Poor neonatal suck+severe hypotonia	Poor neonatal suck and swallow reflexes+ hypertonia	Hypertonia, delayed motor development (1-month)	Hypertonia, delayed gross motor development (4-month), delayed fine motor development (3-month)	Poor swallow reflexes, hypertonia, delayed gross motor development (8-month), delayed fine motor development (6-month)	Poor swallow reflexes, hypertonia, psychomotor regression (5-month)	Poor swallow reflexes, hypertonia, psychomotor regression (5-month)

*NICU, neonatal intensive care unit; Met, methionine; aEEG, amplitude-integratedelectroencephalography; MRI, magnetic resonance imaging; CT, computed tomography*.

## Discussion

When a newborn demonstrates an unexplained feeding problem and a high anion gap metabolic acidosis, doctors will change the direction of diagnosis and treatment to inherited metabolic diseases, especially because in this case, the infant had dystonia, an abnormal MRI, and increased liver enzymes and serum lactate. Even though the MS/MS presented an increased level of 3-methylglutaconic aciduria, we still cannot present a diagnosis because of the various phenotypes present in the neonatal period.

The infant carries two variants of *SERAC1* (c.1502-1G>C from the maternal side, c.227-228dupAT from the paternal side), neither of which have been reported before (http://www.hgmd.cf.ac.uk). She has the typical phenotypes of 3-methylglutaconic acidemia, deafness, and Leigh-like syndrome (present with bilateral basal ganglia lesions on brain MRI) (5). She still has progressive cholestasis and is susceptible to high serum lactate after rehabilitation training and hypoglycemia with low ketone under starving conditions. These phenotypes are substantially different from the transient liver function abnormalities and hypoglycemia reported in the literature (6).

**Figure 3:**
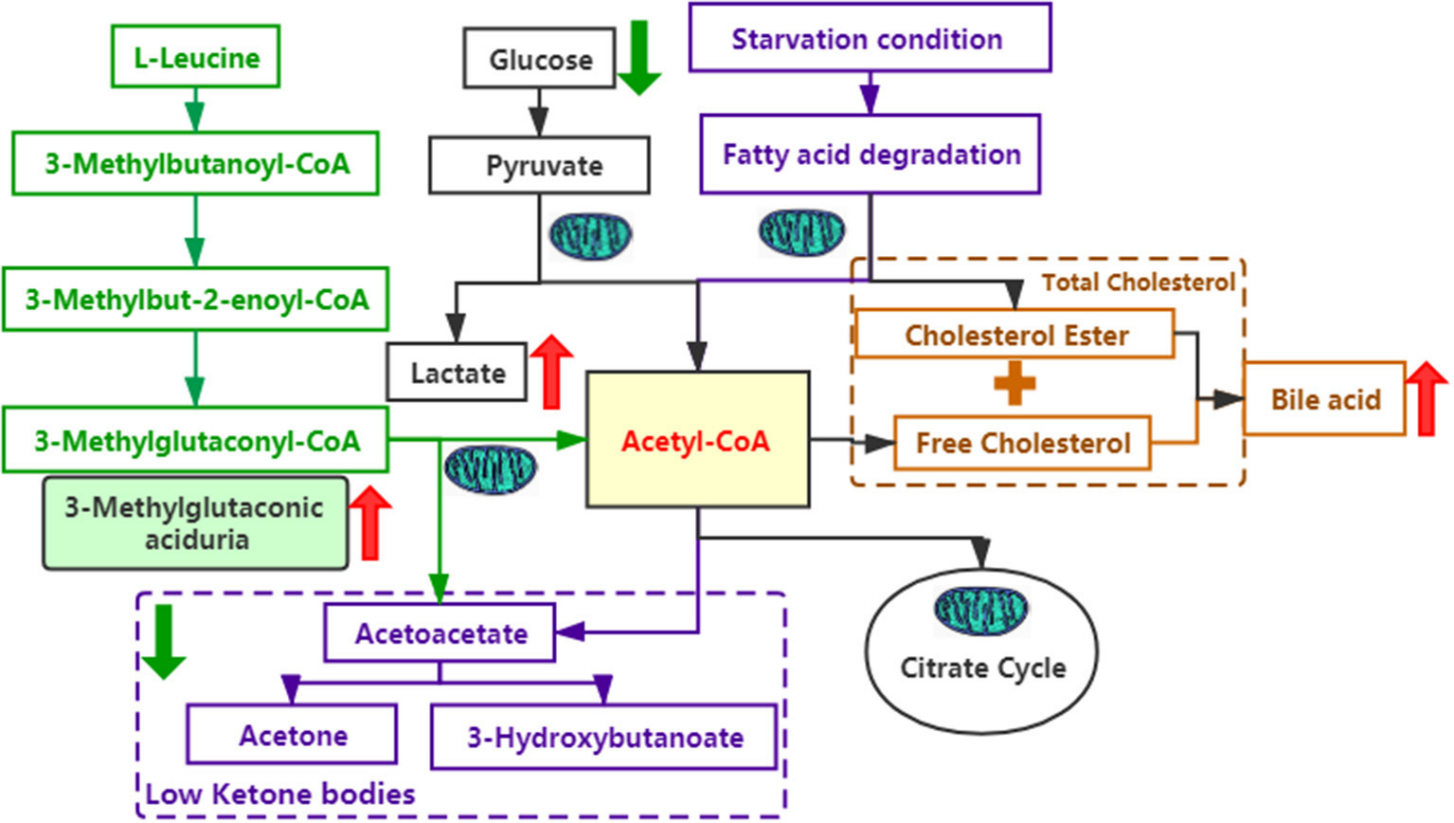
Several metabolic pathways involved in this case. The green font represents the leucine metabolic pathway; the purple font represents the fatty acid metabolic pathway; the black font represents the glucose metabolic pathway; and the yellow font represents the cholesterol metabolic pathway. The red arrows indicate an increase, whereas the green arrows indicate a decrease.

As we know, *SERAC1* encodes for a phosphatidylglycerol remodeler that is essential for mitochondrial functions and intracellular cholesterol trafficking. Although there is no medicine that can completely treat the disease. When the diagnosis is clear, we should give *baclofen* (10 mg, bid), *trihexyphenidyl hydrochloride* (2 mg, bid) to improve her hypertonia and *coenzyme Q10* (30 mg/d), *L-carnitine*(1 g,bid), *vitamin B1* (100 mg/d), and *vitamin E* (100 mg/d) to improve mitochondrial functions. Due to the influence of pandemic of COVID-19, she didn't go to the hospital in time and was not treated until 5 months after birth. But her parents thought there was not obvious effect after treatment and did not adhere to the standard treatment. Here, we try to explain its function through the metabolic pathways (Figure 3). On one hand, mutations in *SERAC1* cause leucine degradation barriers, which, in turn, cause an increase of 3-methylglutaconic aciduria; on the other hand, under glucose deficiency or starvation, fatty acids are oxidized in the liver to produce ketone bodies as energy for the brain. In the process of amino acid metabolism, fatty acid metabolism, and glycolysis, mitochondria are required to participate in the key production of Acetyl-CoA (7). Therefore, low ketone hypoglycemia not only occurs when fatty acid metabolism is abnormal (8) but also when mitochondrial function is abnormal. In the cholesterol metabolism pathway, impaired SERAC1 activity leads to deficiency of PG36:1 and increases free cholesterol (9), which is the main substance for the synthesis of bile acids.

Many syndromes have the phenotype of developmental delay. We were unable to complete the intelligence test because of the patient's deafness and we could not distinguish whether the delayed psychomotor development was caused by mutations in SERAC1 or SATB2 or a combination of the two genes. However, the characteristics of downslanting palpebral fissures, cleft palate, and delayed primary dentition are consistent with phenotypes of SAS because SATB2 is expressed in upper-layer neurons, neural crest progenitors of the jaw, osteoblasts, odontoblasts, and other dental progenitor cells (10). We listed the phenotypes of the two diseases in Supplementary Table 2.

### Strengths and Limitations

We followed-up the case for 15 months and presented a complete process of disease development. These findings extended the phenotype spectrum of the two diseases and provided new insights into such rare diseases. Although medicine of muscle relaxant and antioxidant play roles in symptomatic treatment, the child was not given timely and standardized treatment, which might aggravate her delayed motor development. In addition, MEGDEL syndrome has the phenotype of cholestasis, but we cannot determine whether it is a severe phenotype of MEGDEL syndrome or the superposition of the two rare syndromes.

## Conclusion

We identified two novel variants of *SERAC1* and a *de novo* variant of *SATB2* in a newborn. The case showed typical and specific phenotypes of these two rare diseases, especially the progressive cholestasis and high serum lactate after rehabilitation training and hypoglycemia with low ketone under starving conditions. The article presented a serious phenotype and disease progression, and summarized the metabolic pathways of abnormal indicators in the infant. We hope to enlighten our pediatric colleagues by providing more information on such rare diseases.

## Data Availability Statement

The original contributions presented in the study are included in the article/Supplementary Materials, further inquiries can be directed to the corresponding author/s.

## Ethics Statement

The studies involving human participants were reviewed and approved by The People's Hospital of Xinjiang Uygur Autonomous Region (#2017008). Written informed consent to participate in this study was provided by the participants' legal guardian/next of kin.

## Author Contributions

YS and HZ conceptualized and designed the study, drafted the initial manuscript, and reviewed and revised the manuscript. HW, JY, and BW collected data, carried out the initial analyses, and reviewed and revised the manuscript. LL and WZ designed the data collection instruments, coordinated and supervised data collection, and critically reviewed the manuscript. All authors approved the final manuscript as submitted and agree to be accountable for all aspects of the work.

## Conflict of Interest

The authors declare that the research was conducted in the absence of any commercial or financial relationships that could be construed as a potential conflict of interest.

## Publisher's Note

All claims expressed in this article are solely those of the authors and do not necessarily represent those of their affiliated organizations, or those of the publisher, the editors and the reviewers. Any product that may be evaluated in this article, or claim that may be made by its manufacturer, is not guaranteed or endorsed by the publisher.
